# Phylogeography of Sardinian Cave Salamanders (Genus *Hydromantes*) Is Mainly Determined by Geomorphology

**DOI:** 10.1371/journal.pone.0032332

**Published:** 2012-03-12

**Authors:** Ylenia Chiari, Arie van der Meijden, Mauro Mucedda, João M. Lourenço, Axel Hochkirch, Michael Veith

**Affiliations:** 1 Institut des Sciences de l'Evolution, CNRS-UMR n° 5554, CC 064, Université Montpellier II, Montpellier, France; 2 Centro de Investigação em Biodiversidade e Recursos Genéticos Campus Agrário de Vairão, Vairão, Portugal; 3 Gruppo Speleologico Sassarese, Sassari, Italy; 4 Department of Biogeography, Trier University, Trier, Germany; University of Arkanas, United States of America

## Abstract

Detecting the factors that determine the interruption of gene flow between populations is key to understanding how speciation occurs. In this context, caves are an excellent system for studying processes of colonization, differentiation and speciation, since they represent discrete geographical units often with known geological histories. Here, we asked whether discontinuous calcareous areas and cave systems represent major barriers to gene flow within and among the five species of Sardinian cave salamanders (genus *Hydromantes*) and whether intraspecific genetic structure parallels geographic distance within and among caves. We generated mitochondrial cytochrome *b* gene sequences from 184 individuals representing 48 populations, and used a Bayesian phylogeographic approach to infer possible areas of cladogenesis for these species and reconstruct historical and current dispersal routes among distinct populations. Our results show deep genetic divergence within and among all Sardinian cave salamander species, which can mostly be attributed to the effects of mountains and discontinuities in major calcareous areas and cave systems acting as barriers to gene flow. While these salamander species can also occur outside caves, our results indicate that there is a very poor dispersal of these species between separate cave systems.

## Introduction

Allopatric speciation, the interruption of gene flow between populations, is one of the most widely accepted modes of speciation (e.g., [1], [2], [3]). Therefore, identifying divergent lineages as well as the factors causing interruption of gene flow among populations is key to understanding how species evolve. The detection of distinct genetic units can serve as an initial basis for further investigating both the patterns and processes of speciation (e.g., [4]). Caves are an excellent system for studying processes of colonization, differentiation and speciation because they represent discrete geographical units often with known geological histories. However, phylogenetic relationships among cavernicolous species have rarely been studied (e.g., [5], [6], [7], [8], [9], [10], [11], [12]) because they may be difficult to access and require a higher collecting effort compared to other terrestrial environments. An additional reason could be that few taxa are considered true troglobites (obligate cave species), while many species seem to be troglophiles and might also occur in other similar dark, humid habitat types [13]. In troglophilic taxa, phylogeographic interpretations may be complicated by higher dispersal between caves. Unraveling the interplay of geographic isolation and dispersal should provide additional insights into speciation processes in these ecologically specialized organisms.

Compared to other vertebrate groups, amphibians are commonly considered poor dispersers and are characterized by strong site fidelity [14], [15]. Therefore, significant population structure has generally been observed and current distributions tend to reflect historical events (e.g., glacial refugia, [16], [17], [18]). In a review of intraspecific genetic diversity in amphibians, Vences and Wake [19] highlighted high levels of genetic population structure in relation to geographic variation compared to other vertebrates (see also [20]). Intraspecific genetic diversity in amphibians is often correlated with variation in life-history traits such as reproductive strategies, species distributions, and ecological specializations [19]. Philopatric amphibian species and those with fragmented distributions often show high levels of genetic differentiation between populations (e.g. [21], [22]). However, variation among studies is marked (reviewed in [19]), so that it remains difficult to identify general predictors of genetic population structure and genetic diversity in amphibians.

Plethodontid cave salamanders (genus *Hydromantes*) endemic to Sardinia offer an ideal system to investigate the intra- and interspecific genetic divergence of troglophiles. Adult plethodontid salamanders are lungless, and therefore strongly sensitive to environmental conditions. They require cool and moist environments for optimal breathing through the skin [23]. These salamanders are fully terrestrial, including direct development of eggs laid in terrestrial nests (reviewed in [24]). Sardinian *Hydromantes* are troglophilic organisms so that in addition to caves, they can also be found in other humid environments [25]. When epigean conditions become detrimental (e.g., too hot, too dry, too cold), they can easily access hypogean habitats [24]. For this reason, calcareous areas are an ideal substrate due to the presence of fissures, sinkholes and caverns which provide easy access to underground refugia. Studies of territorial plethodontid salamanders have shown home ranges of a few square meters, which has also been confirmed for mainland Italian *Hydromantes* [26]. All these characteristics suggest that high levels of intraspecific genetic divergence are expected in these organisms.

We here investigate inter- and intraspecific genetic variation in Sardinian cave salamanders using samples from the entire distribution range of all five Sardinian species. Our work consists of a much larger sample size than previous genetic studies [27], [28], [29], [30], including many previously unstudied localities. For each species, the sample sites cover separated geomorphological areas which may constitute potential habitat islands within non-calcareous landscapes [9]. Our aim is to unravel the factors constituting barriers to gene flow among populations of these species. We, therefore, sequenced a fragment of the mitochondrial cytochrome *b* gene in order to infer a large scale phylogeography of the Sardinian cave salamanders. We evaluated two predictions: 1) discontinuities between calcareous areas represent the major barrier to gene flow within and among species and/or 2) intraspecific genetic structure parallels geographic distances. A Bayesian phylogeography approach was applied to infer possible area(s) of cladogenesis for the Sardinian species and to infer the historical and current dispersal routes among distinct populations.

## Materials and Methods

### Ethics Statement

Animals used in this study were not sacrificed and were all released at the sampling sites after sampling. Sampling permits were obtained prior to field work and tissue sampling followed sampling permit requirements.

### Study system

The European *Hydromantes* (subgenera *Speleomantes* Dubois, 1984 and *Atylodes* Temminck & Schlegel, 1838, nomenclature following [31]) (the extra-European species occurring in North America) include eight species distributed in France, northern and central Italy and on the Island of Sardinia. *Hydromantes italicus*, *H. ambrosii*, and *H. strinatii* occur in mainland Italy, while *H. flavus*, *H. supramontis*, *H. sarrabusensis* (*sensu* [29]), *H. imperialis*, and *H. genei* inhabit Sardinia. Previous genetic studies including Sardinian *Hydromantes* were performed with a multilocus approach using allozymes, mitochondrial or nuclear and mitochondrial markers, and mostly focused on the phylogeny, biogeography, and taxonomy of the European species (e.g., [27], [28], [29], [30], [32]; see also [24]). Phylogeographic and population genetic studies including fine sampling of the species range of distributions are still missing. The biogeography of these species is still highly debated due to the poorly supported phylogenetic relationships between species (see [24] for an in-depth review on this subject). This may have resulted from cladogenetic events occurring in a short period of time, and also because of the lack of a good fossil record for these animals in Europe. With the exception of a vertebral fossil from the Middle Miocene (around 13.75 mya) found in Slovakia [33], which has been assigned, but it is still debated to be a plethodontid salamander (see [29]), the fossil record of European plethodontid salamanders is scarce [34], [35]. Due to this, Lanza et al. [24] suggested that it is currently impossible to produce any convincing hypothesis concerning the biogeography of the European *Hydromantes*.

All phylogenetic analyses using different genetic markers have always, however, recovered *H. genei* as a sister taxon to the other European species [27], [29], [30], [32]. High levels of genetic divergence were found among all recognized Sardinian species and within *H. genei* [27], [28], [29], [30], *H. supramontis*, and *H. imperialis* [28], [30]. It has been proposed ([29], and reviewed in [24]) that two separate colonization events were responsible for the current Sardinian *Hydromantes* fauna. An alternative biogeographic hypothesis suggested that *Hydromantes* were once more widespread in Europe. According to this scenario, the Sardo-Corsican microplate (separated from the continent around 33 mya; reviewed in [36]) represented a region of persistence for ancient lineages that once occurred on the European mainland. Therefore, the split between the two main Sardinian clades (a South-Western clade consisting of *H. genei* and an Eastern clade consisting of the other Sardinian species) was a result of a vicariant event associated with the ancient microplate that made up the island. At a later date, but before the cladogenesis of the Eastern clade, *Hydromantes* invaded mainland Italy.

### Sampling and molecular methods

**Figure 1:**
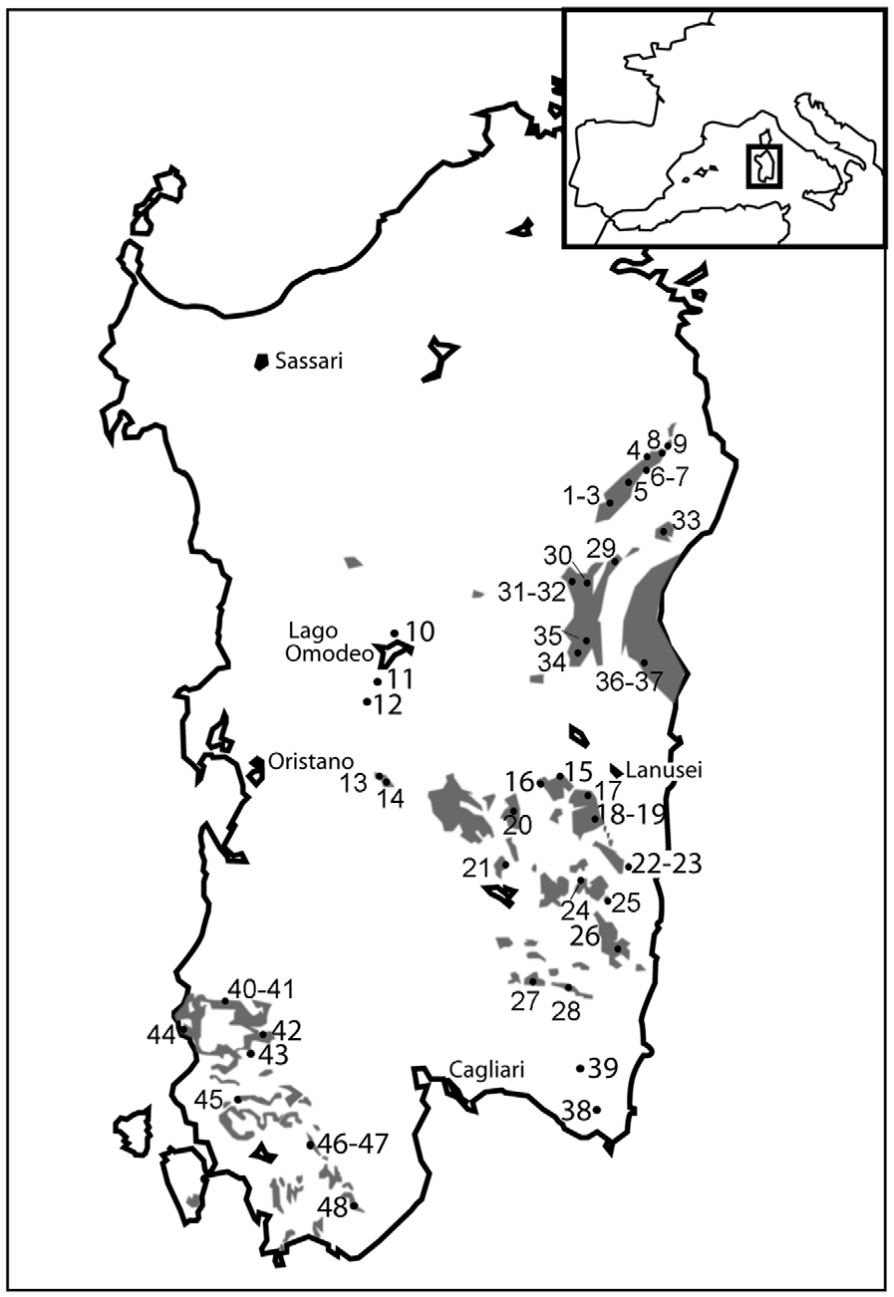
Map of sampling sites in Sardinia. Sampling localities are indicated with numbers corresponding to populations described in Table 1. The smaller insert, top right, indicates the geographic location of Sardinia Island. Populations: 1–9, *H. flavus*; 10–28, *H. imperialis*; 29–37, *H. supramontis*; 38–39, *H. sarrabusensis*; 40–48, *H. genei*. Localities 18–19, 22–23, 40–41, and 46–47 are indicated together due to the small size of the map, but they are separated from each other by several km. Major calcareous areas of the island are indicated in grey.

Sampling was carried out in Sardinia in 2007 and 2008. Sampling localities are indicated in Figure 1 and Table 1 and span the entire known distribution area of each species. We sampled a total of 184 individuals from 48 populations (Table 1), 39 of which (six *flavus*, nine *supramontis*, three *sarrabusensis*, 14 *imperialis*, and seven *genei*, corresponding to a total of 21% of the samples used in the current study and to 15 populations) were already included in a previous study [30]. Geographic coordinates and information about the sampling sites (cave or quarry or deeply under stones) were recorded for each individual. Tail tips were collected and preserved in 99% ethanol. DNA extraction, cytochrome *b* fragment PCR amplification, sequencing, and chromatogram checking are described in [30]. We selected the cytochrome *b* gene as a marker for this study due to the availability of this marker for other species (the outgroups used in this study as well as mainland European *Hydromantes*) for which we did not have tissue samples and because in plethodontids, the cytochrome *b* marker has been observed to have faster rates of molecular evolution than other mitochondrial genes [37]. Purified PCR products were sequenced in both directions, and randomly chosen haplotypes of divergent samples (as from the haplotype network analysis) were sequenced again for confirmation. Amino acid sequence alignment was done in MEGA 4 [38] and then checked by eye. Sequences obtained for this study were deposited in GenBank (accession numbers: JQ582127–JQ582271). Two separate datasets were used for the phylogenetic and population genetic analyses (see below).

**Table 1:** Sampling localities, sample sizes, and summary of genetic data.

Species	Population (pop #)	*N*	*bp*	*h*	*#Mut*	*S*	*Hd*	*π*	*k*
*H. flavus*	Total	32	496	10	26	26	0.879±0.03	0.017±0.01	8.532±4.05
	Lula, Monte Albo- Grotta Conca 'e Crapa (1)	3		1	0	0	0.000±0.00	0.000±0.00	0.000±0.00
	Lula, Monte Albo- Grotta di Nurai (2)	4		1	0	0	0.000±0.00	0.000±0.00	0.000±0.00
	Lula, Monte Albo- Voragine su Saccu (3)	2		1	0	0	0.000±0.00	0.000±0.00	0.000±0.00
	Lula, Monte Albo- Grotta Pupa Niedda (4)	2		2	3	3	1.000±0.50	0.006±0.01	3.000±2.44
	Lula, Monte Albo- Badde Viola (5) *	5		1	0	0	0.000±0.00	0.000±0.00	0.000±0.00
	Siniscola, Monte Albo- Locoli (6) *	4		1	0	0	0.000±0.00	0.000±0.00	0.000±0.00
	Siniscola, Monte Albo- Grotta dell'Inghiottitoio di Locoli (7)	4		3	3	3	0.833±0.22	0.003±0.00	1.667±1.22
	Siniscola, Monte Albo- Nurra di Fruncu 'e Oche (8)	4		2	1	1	0.500±0.27	0.001±0.00	0.500±0.52
	Siniscola, Badde Ghiramonte (9) *	4		1	0	0	0.000±0.00	0.000±0.00	0.000±0.00
*H. supramontis*	Total	42	572	11	51	50	0.877±0.02	0.032±0.02	18.433±8.34
	Dorgali, Monte Coazza- Grotta Gurennoro (29)	5		3	2	2	0.700±0.22	0.002±0.00	1.000±0.80
	Oliena, Lanaitto- Grotta su Bentu (30)	8		2	1	1	0.250±0.18	0.000±0.00	0.250±0.31
	Oliena, Punta sos Nidos- Nurra de sas Palumbas (31)	2		1	0	0	0.000±0.00	0.000±0.00	0.000±0.00
	Oliena, Scala 'e Pradu- Pozzetto di Scala 'e Pradu (32)	5		2	1	1	0.400±0.24	0.001±0.00	0.400±0.44
	Galtelli, Monte Tuttavista- Pozzo 1 di Tres Puntas (33)	5		1	0	0	0.000±0.00	0.000±0.00	0.000±0.00
	Urzulei, Tuvoddoli- Grotta Nurra Tuvodduli (34)	4		1	0	0	0.000±0.00	0.000±0.00	0.000±0.00
	Urzulei- Punta Cuccuttos (35)	3		1	0	0	0.000±0.00	0.000±0.00	0.000±0.00
	Baunei- Grotta ‘Nurra su Sirbone (36) and *	4		1	0	0	0.000±0.00	0.000±0.00	0.000±0.00
	Baunei, Tentinole’- Inghiottitoio di Su Canale (37)	6		2	1	1	0.333±0.22	0.001±0.00	0.333±0.38
*H. sarrabusensis*	Total	10	508	3	3	3	0.644±0.10	0.003±0.00	1.622±1.04
	Castadias, Monte Minniminni (38) *	4		1	0	0	0.000±0.00	0.000±0.00	0.000±0.00
	Burcei, Monte Settefratelli- Sorgente 3 Tulinu (39)	6		2	1	1	0.333±0.22	0.001±0.00	0.333±0.38
*H. imperialis*	Total	76	477	28	61	58	0.963±0.01	0.030±0.02	14.445±6.54
	Sedilo- Funtana Zia Rega (10)	4		2	2	2	0.500±0.27	0.002±0.00	1.000±0.83
	Ardauli- Ponte Canale (11) *	2		2	1	1	1.000±0.50	0.002±0.003	1.000±1.00
	Ula Tirso- Lago Omodeo (12) *	7		2	3	3	0.476±0.17	0.003±0.002	1.429±0.99
	Samugheo, Castello di Medusa- Grotta degli Spelerpes (13)	5		1	0	0	0.000±0.00	0.000±0.00	0.000±0.00
	Asuni, Abba Suergiu- Grotta Stampu de Muscione (14)	4		1	0	0	0.000±0.00	0.000±0.00	0.000±0.00
	Seui, Monte Tonneri- Grotta Sa Muragessa (15)	4		2	1	1	0.500±0.27	0.001±0.001	0.500±0.52
	Seui, Funtana 'e Pauli- Grutta 'e Pauli (16)	5		2	1	1	0.400±0.24	0.001±0.00	0.400±0.44
	Gairo, Taquisara- Grotta di Taquisara (17)	5		2	1	1	0.400±0.24	0.001±0.00	0.400±0.44
	Osini, Serra di Orroli- Grotta di Orroli (18)	6		3	3	3	0.733±0.16	0.004±0.00	1.733±1.17
	Ulassai, Truculu- Grotta de Is Lianas and along the road before and after the Grotta de Is Lianas (19) and *	6		3	4	4	0.600±0.22	0.004±0.00	1.933±1.27
	Sadali, Foresta di Addoli- Near Grotta Margiani Ghiani (20) *	2		1	0	0	0.000±0.00	0.000±0.00	0.000±0.00
	Nurri, Crabarida- Grotta Asuta 'e Scracca (21)	1		-	-	-	-	-	-
	Tertenia- Bosco di San Pietro (22) *	2		1	0	0	0.000±0.00	0.000±0.00	0.000±0.00
	Tertenia- Aqueduct S'ena e Gabudu (23)	2		1	0	0	0.000±0.00	0.000±0.00	0.000±0.00
	Perdasdefugu, Su Sarmentargiu- Grotta Sa Rutta e' Linus (24)	5		2	1	1	0.600±0.18	0.001±0.00	0.600±0.56
	Perdasdefugu, Se Tomeu- Near Grotta 6 Se Tomeu (25) *	3		1	0	0	0.000±0.00	0.000±0.00	0.000±0.00
	Villaputzu, Suergiu- Fossa de Suergiu (26)	3		1	0	0	0.000±0.00	0.000±0.00	0.000±0.00
	San Nicolo Gerrei, Monte Taccu- Grotta Risorgenza Sa Turru (27)	5		2	1	1	0.600±0.18	0.001±0.00	0.600±0.56
	Villasalto, Pardu- Grotta Sa Rutta 'e Scusi (28)	5		2	2	2	0.400±0.24	0.002±0.00	0.800±0.68
*H. genei*	Total	24	490	13	64	61	0.924±0.03	0.037± 0.02	18.159±8.35
	Fluminimaggiore - Mine di Terras Nieddas (40)	5		2	1	1	0.400±0.24	0.001±0.00	0.400±0.44
	Fluminimaggiore - Grotta su Mannau (41)	1		-	-	-	-	-	-
	Domusnovas - Mine Su Corovau (42)	4		3	2	2	0.833±0.22	0.002±0.00	1.000±0.83
	Iglesias - Near Grotta Cuccuru Tiria (43) *	1		-	-	-	-	-	-
	Iglesias - Grotta Gutturu 'e Sattu (44)	2		2	1	1	0.000±0.00	0.000±0.00	0.000±0.00
	Carbonia- Monte Tasua (45) *	5		2	2	2	0.600±0.18	0.002±0.00	1.200±0.91
	Nuxis, Tattinu - Grotta dei geotritoni (46)	4		2	2	2	0.500±0.27	0.002±0.00	1.000±0.83
	Nuxis, Tattinu - La Cava Romana (47)	1		-	-	-	-	-	-
	Domus de Maria, Orbai - Galleria Mazzini (48)	1		-	-	-	-	-	-
**Overall**		184		65					

*Pop #* refers to population number as indicated in Figure 1; *N* indicates the sample size for each species and population, *bp* the number of base pairs used after removal of sites with missing or ambiguous characters in at least one of the sequences (see Materials and Methods), *h* the number of haplotypes, *#Mut* the number of mutations, *S* the number of polymorphic sites, *Hd* the haplotype diversity, π the nucleotide diversity, *k* the mean number of pairwise differences between sequences. *Hd*, *π*, and *k* has been calculated only for populations with two or more individuals. Populations are indicated with the municipality name first followed by the population name. “*” next to the population names indicates that animals were sampled in old quarries, outside caves or deeply buried under stones. No “*” indicates that populations were sampled in caves.

### Phylogenetic analyses

A total of 511 base pairs (bp) of the cytochrome *b* gene common to all species and with less than 5% of missing data for 83 haplotypes (73 and 10 haplotypes from Sardinian and mainland Italian species, respectively) and five outgroups were used for phylogenetic analyses. Cytochrome *b* sequences of the mainland Italian species were from [30]. Haplotypes were obtained by running TCS v1.21 [39] on the cytochrome *b* sequences of each Sardinian and mainland species separately, and then by combining all haplotypes to account for species introgression (which is known to occur among mainland species, see [30]). *Hydromantes brunus* (GenBank accession # AY728234), *H. shastae* (accession # U89611), *H. platycephalus* (accession # U89612), *Ensatina eschscholtzii* (accession # FJ151870), and *Salamandra salamandra* (accession # AY035819) were used as outgroups. The software DAMBE was used to test for saturation at the third codon position [40]. Phylogenetic reconstruction was done by using Maximum Likelihood (ML) and Bayesian Inference (BI). The best fitting model of sequence evolution (GTR+I+G with gamma shape parameter = 1.1868 and proportion of invariable sites = 0.4831) for both analyses was obtained under the AIC criterion with Modeltest 3.7 [41]. Maximum Likelihood analysis was run in PhyML ver. 2.4.4 [42], with 1000 bootstrap replicates. Bayesian inference was conducted with MrBayes 3.1.2 [43]. with 5 million generations, sampling trees every 100th generation, and calculating the consensus tree after excluding the first 5000 sampled trees. Log likelihood scores for the remaining trees were graphed in Tracer 1.5 (http://beast.bio.ed.ac.uk/Tracer) and checked for convergence and appropriateness of the burn in period. Uncorrected genetic p-distances were computed in MEGA [38].

### Bayesian Phylogeography

Three distinct time estimates for the divergence between American and European *Hydromantes* are currently available [29], [37], [44]. These estimates differ one from the other by at least 20 mya (but for [37] and [44] the 95% confidence intervals overlap). While correctly dating the cladogenetic events described in this study would improve the interpretation of the climatic and geological factors correlated with these events, the data and sampling used here and the lack of a better fossil record for the level of divergence do not allow us to perform an accurate dating analysis. We discuss the problems with dating estimates for our data and estimates of the rates of molecular evolution using different estimates of the divergence time between American and European *Hydromantes* as a calibration point in the Information S1.

The Bayesian phylogeographic analysis was run in BEAST v1.6.1 [45] by applying the continuous diffusion model using relaxed random walks following [46] and the related web-available tutorial. We applied the continuous diffusion model instead of the discrete model [47] due to the high number of variables (sampling localities) versus the low number of haplotypes in our dataset. The Bayesian phylogeographic analysis allows inference of where (and when, if some dating is also used) the ancestors of the studied species and populations existed and their phylogeographic history by applying a spatial diffusion approach [48]. An advantage of this method is that geographical space can be divided in infinitesimal regions and dispersal between regions can be modeled as a continuous-time Markov chain (see [46], [48] for more detailed explanations). Furthermore, this kind of analysis is independent, except for the geographic coordinates, from population information such as selection, population size, migration, generation time, etc., which in the case of the species under study are either unknown or are difficult to estimate.

**Figure 2:**
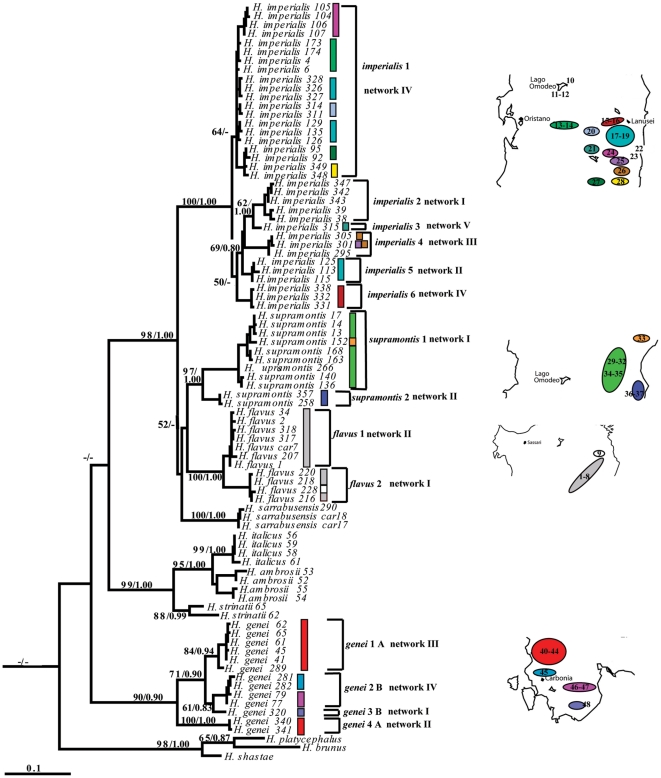
Phylogenetic reconstruction. Phylogenetic Maximum Likelihood tree with bootstrap support and Bayesian posterior probabilities indicated at the nodes. Divergent clades within each species further investigated for genetic distances are indicated with the species name and the clade number next to the tree. Network correspondence (as in Figures 5 and 6) is indicated next to each clade. Differently colored bars next to the haplotype names indicate distinct calcareous areas as shown in the maps next to the tree. Numbers on the maps refer to sampling localities (Table 1). Bootstrap values and posterior probabilities below 50 and 0.7, respectively and outgroups are not shown.

We ran the Bayesian phylogeography analysis only on species most likely belonging to the same cladogenetic event (*H. flavus*, *H. supramontis*, *H. imperialis*, *H. sarrabusensis*) to avoid confounding effects due to the mixture of two separate cladogenetic events (see “Study system” and Discussion for further explanations). A ML unrooted tree was obtained as described above using 511 bp of the cytochrome *b* and all the haplotypes belonging to distinct populations of the four analyzed species (69 haplotypes). This tree was used as a starting tree for the Bayesian phylogeographic analysis [46], [48]. We did not fix the root of the starting tree to avoid biasing the estimates of past geographic locations and of the origin of the studied cladogenetic events. The Bayesian phylogeographic analysis allowed us to infer the area of origin of cladogenesis for the Sardinian *Hydromantes* belonging to the Eastern clade and the dispersal routes within and among these species. Due to the uncertainty associated with the available calibration points for the most accurate dating of our data (see Information S1 for additional information) and due to the poor dating that would result from using divergence time estimates from other studies [49], we choose to not to time-calibrate the Bayesian phylogeographic analysis. We used BEAST for the Bayesian phylogeographic analysis to run five separate chains with 50 million generations, sampling every 5000 generations. We used the Yule model of speciation. Clades and monophyly of each species were fixed. We did not fix the ML groups *imperialis* 1 and *imperialis* 6 (as in Figure 2) as monophyletic because samples belonging to these groups were recovered together on the base of the statistical parsimony-based network analysis (see below). The best model of evolution for this dataset (TIM+I+G) was obtained as described above. Starting values for the precision matrix parameters (1 and 0.5) were chosen as suggested by Lemey (pers. comm.). Because coordinates of the sampling localities were relatively close, we added random noise to identical coordinates using the jitter option with a parameter of 0.05. The log likelihood scores of each chain run was checked for convergence of the chains in Tracer and the first 5000 trees in each chain were discarded. An MCC tree with branch lengths representing posterior median estimates was built with the remaining trees. The MCC tree obtained under the continuous diffusion model was then used as an input in SPREAD (http://www.kuleuven.be/aidslab/phylogeography/SPREAD.html; [50]) by following the online tutorial to analyze and visualize the reconstruction of past phylogeography. Finally, the .kml file produced containing the dispersion routes paths was visualized in Google Earth v6.0.1 (Google Inc.).

### Genetic diversity and population structure

Population genetic analyses were performed on a species by species level using the maximum fragment length without missing data available for each species (see Table 1). Sequences obtained for each species were collapsed into haplotypes by using DnaSP v5 [51], removing sites containing missing or ambiguous data in at least one of the sequences. Number of haplotypes, mutations, segregating sites, singletons, and variants for segregating sites were calculated in DnaSP and/or Arlequin v3.5.1 [52]. We checked for violation of the infinite sites model by comparing the number of haplotypes obtained for each species with the number of segregating sites. Under the infinite sites model, the maximum number of haplotypes (*h*) for a non-recombining genetic region, e.g. cytochrome *b*, is defined as one plus the number of segregating sites (*S*+1) [53]. If *h* is greater than *S*+1 then convergent mutations are implicated at a subset of sites. Species datasets were used to make a statistical parsimony-based network using TCS [39]. Haplotype diversity (the probability that two randomly chosen haplotypes are different in the population), nucleotide diversity (the probability that two randomly chosen sites are different), and mean number of pairwise differences between sequences (the number of differences between haplotypes taken in pairwise comparison in the population) were calculated with Arlequin [52].

Spatial analysis of molecular variance (SAMOVA) was performed using SAMOVA v1.0 [54]. This method recognizes user-defined groups of populations (*K*) that are geographically adjacent, genetically homogeneous within each group, and maximally differentiated from each other in terms of total genetic variance (high *F_CT_* index). A simulated annealing process for each *K* value was repeated 1023 times for each of 100 sets of initial conditions in order to assure that the final population groups were not affected by the initial configuration. The configuration with the higher *F_CT_* after the independent annealing processes was then retained as the best grouping of populations. *K* values were tested for each species, starting from two until the value for which *F_CT_* reached a plateau. Alternative population groupings (selected on the base of current geomorphological knowledge of Sardinian calcareous areas) were tested by applying an analysis of genetic variance (AMOVA) as implemented in Arlequin [52].

To test for a correlation between geographic and genetic distances, an analysis of Isolation by distance (IBD) was run by using the Isolation by Distance Web Service (IBDWS, [55]), available online (http://ibdws.sdsu.edu/~ibdws/distances.html). Linear geographic distances (measured in Km as a linear distance between two locations on the ground) among populations of the same species were calculated by inserting the geographic coordinates of each sampling site in Google Earth. For each species (with the exception of *H. sarrabusensis*, which occurs only in two localities) 20.000 permutations and population average uncorrected p-distance values were used for this analysis. Population average uncorrected distance was calculated as the average of all the pairwise distances among individuals of two distinct populations (distances calculated in MEGA [38]).

## Results

### Species genetic divergence

Saturation at the 3^rd^ codon position was rejected (data not shown). All species were recovered as monophyletic groups with high bootstrap and posterior probability support by the ML and BI analyses (Figure 2) with no instance of haplotype sharing among the Sardinian species. Most relationships among Sardinian species were not well resolved, with the exception of the node separating *H. imperialis* from the other species (Figure 2). Except for *H. sarrabusensis*, distinct clades were recovered in all Sardinian species, some of which had bootstrap support of 80 percent or higher (two main clades in *H. imperialis*, two in *H. supramontis*, two in *H. flavus*, and two in *H. genei*, Figure 2). The well known distinction between two clades of *H. genei* (*genei* A and B *sensu* [24], [27]) was not supported by our analysis, since *H. genei* A was not recovered as monophyletic in our data (Figure 2, *genei* 1A and *genei* 4A). Uncorrected p-distances among full *Hydromantes* species ranged from 0.065 (between *H. ambrosii* and *H. strinatii*) to 0.178 (between *H. imperialis* and *H. genei*) (Information S2). *H. italicus* and *H. ambrosii* had a minimum distance of 0.045, which could be due to the existence of haplotype sharing between the two species (introgression between the two species was found in [30]; in our current analyses these species were collapsed when sharing the same haplotype and reported as possible instance of introgression). Genetic distances among full Sardinian species were generally higher than distances among mainland species (Information S2). The minimum genetic distance of *H. genei* from any of the Sardinian Eastern species (*H. flavus*, *H. supramontis*, and *H. imperialis*) was almost always higher (>0.123) than the maximum distance among any of the Eastern species (except for *H. supramontis* - *H. imperialis*). Intraspecific p-distances among clades (as indicated in Figure 2) were usually lower than the minimum genetic distance among full species, except for *H. genei* 4A from the other clades of *H. genei* (Information S2). The clade of *H. genei* B (*genei* 2B+*genei* 3B) had a maximum genetic distance from samples belonging to the *H. genei* 1A that was below the minimum distance among full *Hydromantes* species (Information S2).

**Figure 3:**
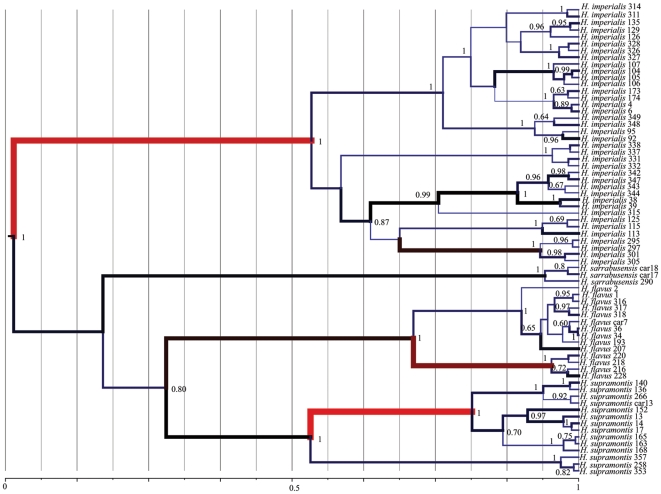
Bayesian phylogeography results. Maximum clade credibility tree from Bayesian phylogeography analysis showing posterior probabilities >0.6 on branch nodes. Color coding and thickness of branches indicate different dispersal rates: thicker and red colored branches correspond to higher dispersal rates, while blue and thinner branches correspond to slower dispersal rates. Numbers at the bottom of the figure indicate relative time scale in percentage from the last common ancestor. “0” indicates that no time has passed and “1” that 100% time has passed since the last common ancestor.

**Figure 4:**
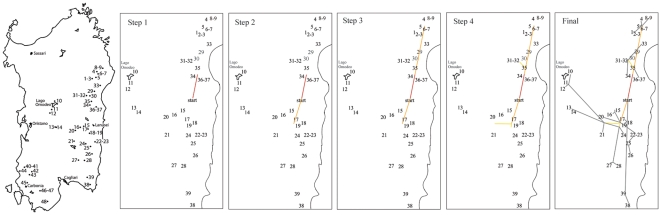
Cladogenesis and dispersal patterns of Sardinian cave salamanders. Origin and dispersal patterns of the species *H*ydromantes *flavus*, *H. supramontis*, *H. imperialis*, and *H. sarrabusensis*. The progressive origin and dispersal patterns - from step 1 (ancestral species, “start” point) to the final/current situation- obtained by Bayesian phylogeographic analysis is indicated from left to right on the map of Sardinia with the localities sampled for the four species (as in Figure 1). Numbers correspond to the same localities coded as in Figure 1. The red-yellow-grey color gradient corresponds to different dispersal rates (from a more rapid to a slower dispersion, respectively).

Bayesian phylogeographic inference supported a rapid dispersal of the ancestor of *supramontis*-*flavus* and *imperialis* (Figures 3 and 4) from an area located in Eastern Sardinia between the current ranges of the *supramontis*-*imperialis* species (Figure 4). Our results indicated changes in the rate of dispersal within and among species, with rapid dispersal non-uniformly distributed across the tree (Figure 3). We obtained a scenario of a rapid dispersal of an ancestral species toward north to give rise to what it is currently recognized as *H. supramontis* (Figure 4, step 1), and then to the *H. imperialis* (Figure 4, step 2). Cladogenesis of an ancestral species into *H. supramontis*, *H. imperialis* and *H. flavus* followed, and according to this dispersion model, a rapid stepping-stone process took place (Figures 3 and 4), with a consequent intraspecific dispersal into multiple adjacent areas (with back migrations as in the case of the *imperialis*) and genetic differentiation of *H. sarrabusensis* in its current distribution range in the South-East of Sardinia (Figure 4, step 4 and final scenario).

### Intraspecific genetic diversity

Between 477 and 572 bp of the cytochrome *b* gene were used to analyze the genetic diversity of the five Sardinian species (Table 1). The total number of haplotypes, mutations and polymorphic sites obtained for each species supported the infinite sites model. The total number of mutations was higher than the number of segregating sites in three out of the five studied species (*H. supramontis*, *H. imperialis*, and *H. genei*) indicating that multiple mutations occurred at some sites. In fact, while in *H. flavus* and *H. sarrabusensis* all the polymorphic sites were two-fold variants, in the other three species between one and three sites were three-fold variants. We obtained a total of 65 haplotypes for five species. Haplotype diversity was highest in *H. imperialis* and *H. genei* and lowest in *H. sarrabusensis* (Table 1). The numbers of mutations and segregating sites were higher in *H. supramontis*, *H. imperialis*, and *H. genei* than in the other two species, resulting in the highest nucleotide diversity and the highest mean number of pairwise differences among sequences (Table 1).

### Population genetic structure

**Figure 5:**
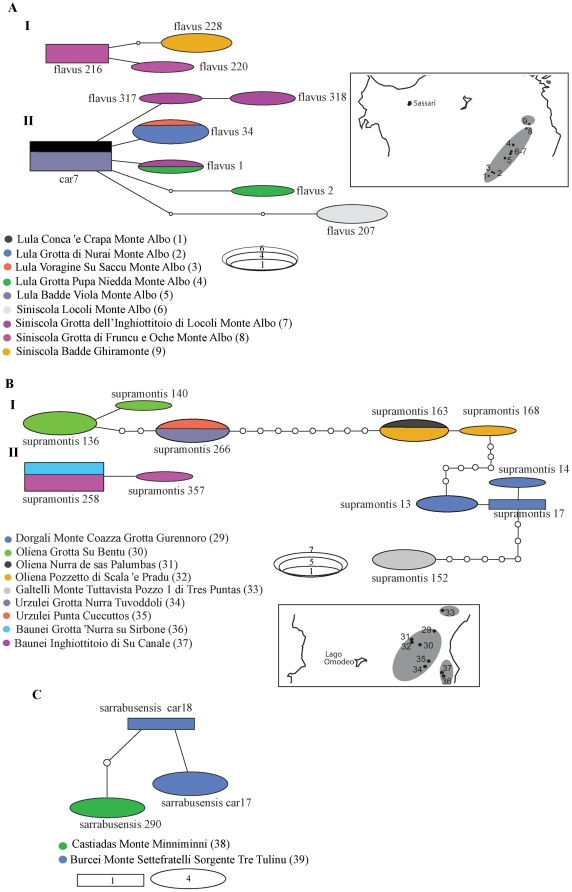
Haplotype networks for Sardinian *Hydromantes* species. A) Haplotype networks of *H. flavus*; B) Haplotype networks of *H. supramontis*; C) Haplotype network of *H. sarrabusensis*. The length of the respective fragment and the number of individuals used for each species are given in Table 1. Numbers in parentheses after the locality name correspond to population numbers as in Figure 1. Small white circles indicate missing haplotypes. Rectangles indicate possible ancestral haplotypes. The size of the rectangles and ellipses is proportional to the number of individuals sharing the same haplotypes as indicated. Colors correspond to distinct localities as indicated in each legend. Small insets next to each species network indicate calcareous massifs groups (circles in grey) according to current geological knowledge for the sampled localities. Localities which are not encircled by grey shadows do not occur in a calcareous area. *H. sarrabusensis* does not occur in a calcareous area.

**Figure 6:**
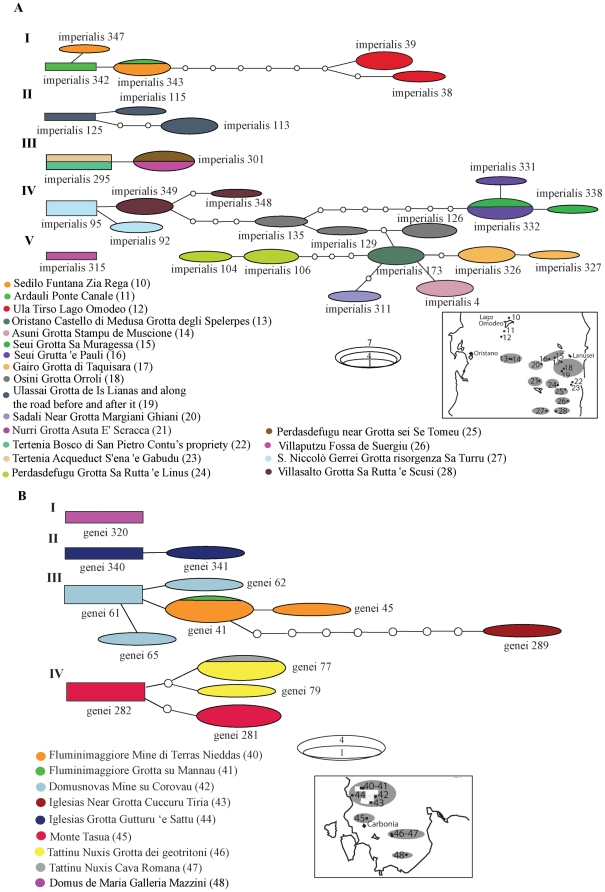
Haplotype networks for Sardinian *Hydromantes* species. A) Haplotype networks of *H. imperialis*, and B) Haplotype networks of *H. genei*. See Figure 5 for details.

Intraspecific subdivision into distinct clades was supported by the haplotype networks (Figures 5 and 6). In all species, haplotype sharing among populations occurred only in a few cases. The majority of the localities contained private haplotypes. In *H. flavus*, individuals from Siniscola were divided among the two haplotype networks (Figure 5a). Within this species, most haplotypes differed just by one or two mutations from the most common one (car7). In *H. supramontis*, the easternmost samples (Baunei) were recovered as a network separate from the rest (Figure 5bII). The main network of this species (Figure 5bI) included samples from all other localities. The network was divided into four main haplotype sub-groups, separated by at least seven or eight mutations. In *H. sarrabusensis*, samples from the two localities differed by at least two mutations (Figure 5c). In *H. imperialis*, five distinct networks were recovered (Figure 6a). Two networks consisted of samples from one locality each (network V: Nurri - locality 21; network II: Ulassai - locality 19) (Figure 6a). Within network I, the samples from Lake Omodeo (locality 12) formed a distinct sub-group, separated from the closest haplotype (samples from localities 10 and 11) by at least seven mutations. The majority of the localities were recovered together in a single network (IV) defined by distinct haplotypes, with no haplotype sharing. Within this network, the samples from localities 15 and 16 constituted a sub-group (recovered as separated clades by the phylogenetic analyses, Figure 2) separated from the closest haplotype (samples from locality 18) by a minimum of eight mutations (Figure 6a). In the *H. genei* haplotype network (Figure 6b) the southernmost sample (locality 48) was recovered as a separate network (network I). The samples from locality 44 (Iglesias), the westernmost locality of the *H. genei* dataset and in the middle of the distribution of the *genei* A, were recovered as a separate network well distinct from all the other samples. The other sample from Iglesias (locality 43, Figure 1), despite being part of the network III, was fairly distinct from the other sampling sites. The networks III and IV contained samples of *H. genei* A and *H. genei* B (*sensu* [24], [27]), respectively. Minimum absolute distances to connect haplotype networks within each species are presented in the Information S3.

**Figure 7:**
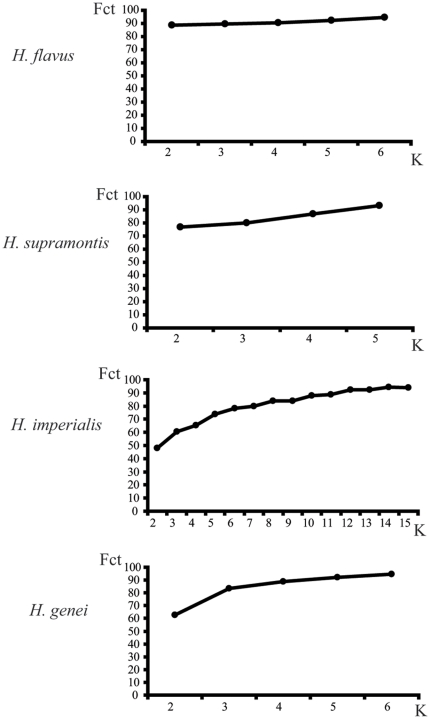
Optimal intraspecific grouping that maximizes genetic divergence. Plot of the number of groupings (*K*, on the x-axes) versus the *F_CT_* values obtained for each *K* grouping (y-axes) according to the SAMOVA results. See text for details.

Significant levels of genetic structure, with high intrapopulation genetic variance, were found within each species (Information S4). When testing each species for the most probable population grouping to maximize genetic variance among groups, the *F_CT_* values obtained on the basis of current geomorphological knowledge of Sardinian calcareous areas were always lower than those obtained by the SAMOVA (Information S4). While in *H. flavus*, *F_CT_* value already reached a plateau for *K* = 2 (with *K* = number of groups tested, Figure 7), three or four clusters better reflected the population structure of *H. genei* and *H. supramontis* (Information S4 and Figure 7). Genetic variance was found to be especially high in *H. imperialis*, where *F_CT_* reached a plateau for *K* of ca. seven to eight groups (Information S4 and Figure 7). In *H. genei*, the two clusters defined as *H. genei* A and *H. genei* B did not maximize the genetic variance among groups (Information S4).

Genetic population structure was found to be correlated, except in one case, with geographic distances (*H. flavus*, p = 0.0126; *H. supramontis* p = 0.0269; *H. imperialis*, p = 0.0038; *H. genei*, p = 0.1145). The correlation coefficient, r, indicated either a strong (*H. flavus*, r = 0.7550, Z = 7.7303), a medium (*H. supramontis*, r = 0.4768, Z = 20.4394), or a weak (*H. imperialis*, r = 0.3027, Z = 209.9705; *H. genei*, r = 0.2753, Z = 41.3510) correlation. In *H. flavus*, two distinct groups with correlated genetic and geographic distances were found (low genetic-geographic distance and high genetic-geographic distances, data not shown). A similar, even if less marked pattern was observed in *H. supramontis*, while in *H. imperialis* and *H. genei* high genetic distances were almost uniformly distributed across geographical distances (data not shown).

## Discussion

### Mountains and calcareous areas as centers of differentiation of Hydromantes species

Our study revealed strong genetic differentiation among populations of *Hydromantes* species, suggesting that gene flow is very low even though these salamanders can live outside of caves. Until now, only a single hypothesis was put forward concerning the patterns and areas of cladogenesis in Sardinian cave salamanders. Cimmaruta et al. [28] attributed the high genetic divergence among species to the influence of paleoclimatic fluctuations during the Quaternary and the use of mountainous areas as refugia. Three major refugia have been proposed [28]: Monte Albo for *H. flavus* (corresponding to the current range of distribution of the species), the ring-shaped area around the Gennargentu (in Figure 1 located among the northern and southern sampling localities of *H. imperialis* and *H. supramontis*, respectively) for *H. supramontis* and *H. imperialis* (including *H. sarrabusensis*, which was elevated to a full species after 1998), and the area of the Iglesiente for *H. genei* (corresponding to the Western part of the current range of distribution of the species). After initial divergence in allopatry, Cimmaruta et al. [28] proposed a scenario with further fragmentation within *H. imperialis* and *H. supramontis* into restricted genetic islands corresponding to separated mountain chains (Oliena-Orgosolo-Urzulei-Baunei mountains – roughly represented by our sampling localities 30, 31, 32, 34, 35-, Dorgali-Baunei- sampling localities 29, 36, 37-, Ogliastra-Barbagia - sampling localities 15, 16, 17, 18, 19, 20-, Gerrei-Sarcidano - sampling locality 21-), followed by few episodes of secondary contacts.

Mountain chains and elevational gradients have been reported to act as barriers to gene flow in amphibians [18], [56], [57], [58] and mountain regions have been recognized as centers of high endemism and diversification for many Sardinian organisms (e.g., [59], [60], [61]). Many biogeographic and phylogeographic studies have included Sardinian organisms (e.g., [62], [63], [64], [65], [66], [67], [68]), but very few of them focused on diversification patterns within this island, often using only a very sparse sampling (but see [28]). For some Sardinian species, intra- and interspecific divergences have been associated with sea-level oscillations (5–2 mya) and the beginning of the Ice Age (around 2 mya) (reviewed in [36]). While our data do not allow us to time the studied cladogenetic events to assess the influence that climate and geological changes may have had on them, our results indicate that major mountain chains seem to be primary areas of species diversification from which other suitable environments (mostly calcareous areas) were either colonized or used as refugia. Two possible scenarios, which could not be further investigated with the current data, could explain our results; 1) a dispersal and colonization scenario from one locality to another, versus 2) a scenario in which these sampled areas were already colonized by an ancestral species and the observed genetic differentiation resulted from an interruption of gene flow between populations and from differences in effective population size. This would affect the rate of neutral divergence among isolated geographic fragments. This latter scenario would, however, require more frequent extinction events of ancestral genetic forms to explain our results.

Our results suggest divergence of an ancestor for Eastern Sardinian species to have occurred near the Gennargentu mountains. Allopatric speciation in separate mountain areas as proposed by Cimmaruta et al. [28] is also supported by our data. However, our results suggest a first split of an ancestor of *H. supramontis-H. flavus* from an ancestor of the other species and further cladogenesis following a stepping-stone mode. Intraspecific divergence can be explained by dispersal events to multiple adjacent areas, with few secondary contacts or by genetic differentiation and consequent extinction of an ancestral genotype among populations with different population size. Only one area of dispersal or of persistence of an ancestral genotype, corresponding to the center of the Monte Albo, was found for *H. flavus*. Dispersal to adjacent areas followed by genetic divergence within this species or genetic differentiation from the ancestral genotype due to habitat isolation and different effective population sizes are also supported by our parsimony-based analysis. A major area of dispersal (or with an ancestral genotype) corresponding to the calcareous massif and to the mountain complex of Oliena-Orgosolo-Urzulei (populations 30–35) was recovered for *H. supramontis*. From there, an initial range expansion to adjacent areas and a secondary stepping-stone migration and genetic differentiation in new localities, including separate calcareous areas and mountains (populations 33 and 36–37), or local genetic differentiation took place. Back migration or persistence of an ancestral genotype are suggested between some of the localities within the same calcareous complex. In *H. imperialis*, the initial diversification probably occurred in the calcareous area and mountain complex of the Ogliastra (populations 17 and 19), from which individuals dispersed to adjacent areas within and to separate calcareous massifs or where genetic differentiation occurred locally. Finally, our results support an early diversification of an ancestor of *H. sarrabusensis* and secondary dispersal of this species to its current distribution area. We cannot, however, exclude genetic differentiation of *H. sarrabusensis* occurring in its current habitat paralleled by the extinction of his ancestral genotype.

Calcareous areas and cave systems have been shown to offer refugia during times of changing environmental conditions and to be associated with genetic divergence and speciation in several taxa (e.g., [9], [10], [11], [69], [70], [71]). In Sardinian cave salamanders, no strong intraspecific divergence was found between animals sampled within caves and outside (under stones or in quarries). However, separate caves and calcareous systems were observed to be correlated to some degree with intraspecific genetic isolation. Hence, although the Sardinian *Hydromantes* also occur outside caves (troglophiles *sensu* [13]), the intraspecific differentiation between discontinuous cave systems was strong. In *H. flavus* and *H. supramontis*, genetic divergence seems to be mostly correlated to geomorphological separation of distinct calcareous massifs, with isolation by distance also playing a role in explaining the observed patterns of divergence. The pattern of genetic divergence was more complex in *H. imperialis* and *H. genei*. In these species, geomorphology seems to explain only part of the genetic divergence (e.g. networks I, III and V for *H. imperialis* and networks I and IV for *H. genei*). However, in both species we also found genetically strongly diverged lineages at a small geographic distance. The reasons for these patterns may be found in local environmental heterogeneity, which might have restricted gene flow, or in a more complex evolutionary history of the populations. In the case of *H. genei*, additional sampling from other sites and further geological investigation may help to identify possible barriers to gene flow. In fact, the strongly divergent samples of *H. genei* represented the westernmost part of its distribution.

Gene flow among Sardinian *Hydromantes* populations (i.e. haplotype sharing among localities) was extremely low and occurred mostly among geographically proximate sites. This suggests that phylogeographic breaks also occur in continuous areas without any obvious long-term geographic barriers. This phenomenon may result from the combination of low dispersal capacity and low population size [72], even if the latter does not seem to be the case for Sardinian salamanders (see also below). However, as our data only represent matrilineal genealogies, we cannot assess sex-biased dispersal with males migrating further than females, as observed in other Urodela [73], [74]. Further studies using multilocus data [12] should improve our understanding of speciation and species delimitation in these species.

### Taxonomic implications

A detailed review of the genus *Hydromantes*, including the extra-European species, is beyond the scope of this article, and was reported by Wake et al. [75]. Nascetti et al. [27] described two genetically distinct clades within *H. genei* called *H. genei* A and *H. genei* B that suggested the existence of cryptic species. This result was also confirmed by later studies [29], [30]. Although our results clearly support two genetically divergent clades within *H. genei*, these do not correspond to the clades defined by Nascetti et al. [27]. Our sampling included new localities of *H. genei* containing two individuals from the westernmost part of its range that were recovered as genetically most divergent. When excluding these samples, genetic distances among clades A and B became similar to the intraspecific genetic divergence in other Sardinian species. Hence, the interpretation of these clades as cryptic species might have been influenced by insufficient knowledge of the intraspecific genetic divergence in other *Hydromantes* species and insufficient sampling of *H. genei*. We agree with Speybroeck et al. [76] who argued that a taxonomic revision of the Sardinian *Hydromantes* may be premature at this time. Further studies with even finer sampling, including populations from potential contact zones among genetically divergent lineages are required.

### Conservation considerations

Plethodontid salamanders are considered excellent biological indicators of environmental change due to their ecological characteristics and life-history traits. Among these are site fidelity and poor dispersal, sensitivity to habitat disturbance and destruction, generally high population density, and longevity (reviewed in [77]). Henle et al. [78] included the European *Hydromantes* species among the amphibians that are likely to suffer most from future climate changes due to the strong environmental and temperature sensitivity. However, cave habitats may be strongly buffered against climate fluctuations in comparison to other habitats. All Sardinian *Hydromantes* species are currently listed in the annexes II and IV of the habitats directive 43/92/EEC, as well as listed as Vulnerable/Lower Risk in the IUCN Red Data book of Italy [79]. For these reasons, Lanza [80] proposed that certain caves should be protected.

Our results revealed the existence of significant genetic divergence within Sardinian salamander species, with the presence of many isolated, genetically unique populations that should be protected. The large genetic diversity observed within each species suggests large population size and a capacity for resilience to climate change. However, the low number of individuals per population we studied does not allow us to infer a more precise conservation evaluation. Therefore, more data on the ecology of these species, as well as a finer population genetic study including faster evolving nuclear markers are urgently required to delineate specific conservation measures.

## Supporting Information

Information S1 **Problems relative to dating estimates for our data.**
(DOC)

Information S2 **Genetic p-distances among species (a) and intraspecific clades (b) of Italian** ***Hydromantes*** **.** Average p-distances (minimum and maximum p-distances in parenthesis) based on 511 bp of the cytochrome *b* gene. Numbers with each species refer to clades as in Figure 2.
(DOC)

Information S3 **Intraspecific haplotype networks distances.** Minimum number of steps (absolute genetic distances) to connect intraspecific single haplotype networks.
(DOC)

Information S4 **Results of the SAMOVA/AMOVA analyses.** Intraspecific genetic structure for each *Hydromantes* species on the base of the within and among population genetic variance, the geographic location and sampling site. This was not tested for *H. sarrabusensis*, as only one haplotype network was recovered for this species. Populations belonging to distinct clusters (gene pools) are indicated in parenthesis with numbers referring to populations as in Table 1. *F_ST_*, *F_CT_*, and *F_SC_* indicate the proportion of total variance among populations, among groups, and among populations within groups, respectively. * indicates significant p-values<0.05.
(DOC)

## References

[1] Mayr E (1942). Systematics and the Origin of Species.

[2] de Queiroz K, Howard DJ, Berlocher SH (1998). The general lineage concept of species, species criteria, and the process of speciation: A conceptual unification and terminological recommendations.. Endless Forms: Species and Speciation.

[3] de Queiroz K (2007). Species concepts and species delimitation.. Syst Biol.

[4] McKinnon JS, Rundle HD (2002). Speciation in nature: the threespine stickleback model systems.. Trends Ecol Evol.

[5] Allegrucci G, Caccone A, Cesaroni D, Sbordoni V (1992). Evolutionary divergence in *Dolichopoda* cave crickets: A comparison of single copy DNA hybridization data with allozymes and morphometric distances.. J Evolution Biol.

[6] Allegrucci G, Trucchi E, Sbordoni V (2011). Testing phylogenetic hypotheses for reconstructing the evolutionary history of *Dolichopoda* cave crickets in the eastern Mediterranean.. J Biogeogr.

[7] Allegrucci G, Rampini M, Gratton P, Todisco V, Sbordoni V (2009). Tempo and mode of species diversification in *Dolichopoda* cave crickets (Orthoptera, Rhaphidophoridae).. Mol Phylogenet Evol.

[8] Hedin MC (1997). Molecular phylogenetics at the population/species interface in cave spiders of the southern Appalachians (Araneae: Nesticidae: *Nesticus*).. Mol Biol Evol.

[9] Veith M, Lipscher E, Öz M, Kiefer A, Baran I (2008). Cracking the nut: Geographical adjacency of sister taxa supports vicariance in a polytomic salamander in the absence of node support.. Mol Phylogenet Evol.

[10] Martinsen L, Venanzetti F, Bachmann L (2009). Phylogeography and mitochondrial DNA divergence in *Dolichopoda* cave crickets (Orthoptera, Rhahidophoridae).. Hereditas.

[11] Niemiller ML, Fitzpatrick BM, Miller BT (2008). Recent divergence with gene flow in Tennessee cave salamanders (Plethodontidae: *Gyrinophilus*) inferred from gene genealogies.. Mol Ecol.

[12] Niemiller ML, Near TJ, Fizpatrick BM (2011). Delimiting species using multilocus data: diagnosing cryptic diversity in the Southern cavefish, *Typlichthys subterraneus* (Teleostei: Amblyopsidae).. Evolution.

[13] Howarth FG (1983). Ecology of cave arthropods.. Annu Rev Entomology.

[14] Beebee TJC (1996). Ecology and conservation of amphibians.

[15] Smith MA, Green DM (2005). Dispersal and the metapopulation paradigm in amphibian ecology and conservation: are all amphibian populations metapopulations?. Ecography.

[16] Steinfartz S, Veith M, Tautz D (2000). Mitochondrial sequence analysis of *Salamandra* taxa suggests old splits of major lineages and postglacial recolonizations of Central Europe from distinct source populations of *Salamandra salamandra*.. Mol Ecol.

[17] Tarkhnishvili DN, Thorpe RS, Arntzen JW (2000). Pre-Pleistocene refugia and differentiation between populations of the Caucasian salamander (*Mertensiella caucasica*).. Mol Phylogenet Evol.

[18] Zeisset I, Beebee TJC (2008). Amphibian phylogeography: a model for understanding historical aspects of species distributions.. Heredity.

[19] Vences M, Wake DB, Heatwole HH, Tyler M (2007). Speciation, species boundaries and phylogeography of amphibians.. Amphibian Biology, vol. 6.

[20] Nevo E, Beiles A, Ben-Shlomo R (1984). The evolutionary significance of genetic diversity: ecological, demographic and life history correlates.. Lecture Notes in Biomathematics.

[21] Larson A, Wake DB, Yanev KP (1984). Measuring gene flow among populations having high levels of genetic fragmentation.. Genetics.

[22] Crottini A, Chiari Y, Mercurio V, Meyer A, Vences M (2008). Into the canyons: the phylogeography of the Malagasy frogs *Mantella expectata* and *Scaphiophryne gottlebei* in the arid Isalo Massif, and its significance for conservation (Amphibia: Mantellidae and Microhylidae).. Org Divers Evol.

[23] Noble GK (1931). The biology of amphibian.

[24] Lanza B, Pastorelli C, Laghi P, Cimmaruta R (2005). A review of systematics, taxonomy, genetics, biogeography and natural history of the genus *Speleomantes* Dubois, 1984 (Amphibia Caudata Plethodontidae).. Atti Mus Stor Nat Trieste (Suppl.).

[25] Voesenek LACJ, van Rooy PTJC, Strijbosch (1987). Some autecological data on urodeles of Sardinia.. Amphibia-Reptilia.

[26] Salvidio S, Lattes A, Tavano M, Melodia F, Pastorino MV (1994). Ecology of a *Speleomantes ambrosii* population inhabiting an artificial tunnel.. Amphibia-Reptilia.

[27] Nascetti G, Cimmaruta R, Lanza B, Bullini L (1996). Molecular taxonomy of European plethodontid salamanders (genus *Hydromantes*).. J Herpetol.

[28] Cimmaruta R, Nascetti G, Forti G, Lanza B, Bullini L (1998). Paleogeografia della Sardegna ed evoluzione degli *Hydromantes* (Amphibia, Plethodontidae).. Biogeographia.

[29] Carranza S, Romano A, Arnold EN, Sotgiu G (2007). Biogeography and evolution of European cave salamanders, *Hydromantes* (Urodela: Plethodontidae), inferred from mtDNA sequences.. J Biogeogr.

[30] Van der Meijden A, Chiari Y, Mucedda M, Carranza S, Corti C (2009). Phylogenetic relationships of Sardinian cave salamanders, genus *Hydromantes*, based on mitochondrial and nuclear DNA sequence data.. Mol Phylogenet Evol.

[31] Vieites DR, Nieto Roman S, Wake MH, Wake DB (2011). A multigenic perspective on phylogenetic relationships in the largest family of salamanders, the Plethodontidae.. Mol Phylogenet Evol.

[32] Pyron RA, Wiens JJ (2011). A large-scale phylogeny of Amphibia including over 2800 species, and a revised classification of extant frogs, salamanders, and caecilians.. Mol Phylogenet Evol.

[33] Venczel M, Sanchíz B (2005). A fossil plethodontid salamander from the Middle Miocene of Slovakia (Caudata, Plethodontidae).. Amphibia-Reptilia.

[34] Delfino M, Razzetti E, Salvidio S, Salvidio S, Pastorino MV (2002). Pletodontidi europei: dati paleontologici e considerazioni biogeografiche.. Primo Convegno Nazionale «Biologia dei Geotritoni europei. Genere *Speleomantes*», Programma e Riassunti.

[35] Delfino M, Razzetti E, Salvidio S, Salvidio S, Poggi R, Doria G, Pastorino MV (2005). European plethodontids: palaeontological data and biogeographical considerations (Amphibia).. Atti del Primo Convegno Nazionale «Biologia dei geotritoni europei. Genere *Speleomantes*». Annali Mus. civ. St. nat. G. Doria, Genova, Italy.

[36] Grill A, Casula P, Lecis R, Menken S, Weiss S, Ferrand N (2007). Endemism in Sardinia.. Phylogeography of Southern European Refugia. Evolutionary perspectives on the origins and conservation of European biodiversity.

[37] Mueller RL (2006). Evolutionary rates, divergence dates, and the performance of mitochondrial genes in Bayesian Phylogenetic Analysis.. Syst Biol.

[38] Tamura K, Dudley J, Nei M, Kumar S (2007). MEGA: Molecular Evolutionary Genetic Analysis (MEGA) software version 4.0.. Mol Biol Evol.

[39] Clement M, Posada D, Crandall K (2000). TCS: a computer program to estimate gene genealogies.. Mol Ecol.

[40] Xia X, Xie Z, Salemi M, Chen L, Wang Y (2003). An index of substitution saturation and its application.. Mol Phylogenet Evol.

[41] Posada D, Crandall KA (1998). Modeltest: testing the model of DNA substitution.. Bioinformatics.

[42] Guindon S, Gascuel O (2003). A simple, fast, and accurate algorithm to estimate large phylogenies by maximum likelihood.. Syst Biol.

[43] Huelsenbeck JP, Ronquist F (2001). MRBAYES: Bayesian inference of phylogenetic trees.. Bioinformatics.

[44] Vieites DR, Min M-S, Wake DB (2007). Rapid diversification and dispersal during periods of global warming by plethodontid salamanders.. Proc Natl Acad Sci.

[45] Drummond AJ, Rambaut A (2007). BEAST: Bayesian evolutionary analysis by sampling trees.. BMC Evol Biol.

[46] Lemey P, Rambaud A, Welch JJ, Suchard MA (2010). Phylogeography takes a relaxed random walk in continuous space and time.. Mol Biol Evol.

[47] Lemey P, Rambaud A, Dummond AJ, Suchard MA (2009). Bayesian phylogeography finds its roots.. PLoS Computational Biology.

[48] Bloomquist EW, Lemey P, Suchard MA (2010). Three roads diverged? Routes to phylogeographic inference.. Trends Ecol Evol.

[49] Graur D, Martin W (2004). Reading the entrails of chickens: molecular timescales of evolution and the illusion of precision.. Trends in Genetics.

[50] Bielejec F, Rambaut A, Suchard MA, Lemey P (2011). SPREAD: Spatial Phylogenetic Reconstruction of Evolutionary Dynamics.. Bioinformatics.

[51] Librado P, Rozas J (2009). DnaSP v5: A software for comprehensive analysis of DNA polymorphism data.. Bioinformatics.

[52] Excoffier L, Lischer HEL (2010). Arlequin suite ver. 3.5: A new series of programs to perform population genetics analyses under Linux and Windows.. Mol Ecol Res.

[53] Depaulis F, Veuille M (1998). Neutrality tests based on the distribution of haplotypes under an infinite-site model.. Mol Biol Evol.

[54] Dupanloup I, Schneider S, Excoffier L (2002). A simulated annealing approach to define the genetic structure of populations.. Mol Ecol.

[55] Jensen JL, Bohonak AJ, Kelley ST (2005). Isolation by distance, web service.. BMC Genetics.

[56] Funk WC, Blouin M, Corn PS, Maxell BA, Pilliod DS (2005). Population structure of Columbia spotted frogs (*Rana luteiventris*) is strongly affected by the landscape.. Mol Ecol.

[57] Shepard DB, Burbrink FT (2009). Phylogeographic and demographic effects of Pleistocene climatic fluctuations in a montane salamander, *Plethodon fourchensis*.. Mol Ecol.

[58] Parra-Olea G, Windfield JC, Velo-Antón G, Zamudio KR (2011). Isolation in habitat refugia promotes rapid diversification in a montane tropical salamander.. J Biogeogr.

[59] Caccone A, Sbordoni V (2001). Mitochondrial biogeography of cave life: a study using mitochondrial DNA from Bathysciine beetles.. Evolution.

[60] Grill A, Raijmann LEL, van Ginkel W, Gkioka E, Menken SBJ (2007). Genetic differentiation and natural hybridization between the Sardinian endemic *Maniola nurag* and the European *Maniola jurtina*.. J Evol Biol.

[61] Lecis R, Norris K (2004). Population genetic diversity of the endemic Sardinian newt *Euproctus platycephalus*.. Biol Conserv.

[62] Capula M (1996). Evolutionary genetics of the insular lacertid lizard *Podarcis tiliguerta*: genetic structure and population heterogeneity in a geographically fragmented species.. Heredity.

[63] Ketmaier V, Argano R, Caccone A (2003). Phylogeography and molecular rates of subterranean aquatic Stenasellid isopods with a peri-Tyrrhenian distribution.. Mol Ecol.

[64] Harris DJ, Pinho C, Carretero MA, Corti C, Böheme W (2005). Determination of genetic diversity within the insular lizard *Podarcis tiliguerta* using mtDNA sequence data, with a reassessment of the phylogeny of *Podarcis*.. Amphibia-Reptilia.

[65] Biollaz F, Bruyndockx N, Beuneux G, Mucedda M, Goudet J (2010). Genetic isolation of insular populations of the Maghrebian bat, *Myotis punicus*, in the Mediterranean Basin.. J Biogeogr.

[66] Ribera I, Fresneda J, Bucur R, Izquierdo A, Vogler A (2010). Ancient origin of a Western Mediterranean radiation of subterranean beetles.. BMC Evol Biol.

[67] Salvi D, Harris DJ, Bombi P, Carretero MA, Bologna MA (2010). Mitochondrial phylogeography of the Bedriaga's rock lizard, *Archeolacerta bedriagae* (Reptilia: Lacertidae) endemic to Corsica and Sardinia.. Mol Phylogenet Evol.

[68] Bisconti R, Canestrelli D, Nascetti G (2011). Genetic diversity and evolutionary history of the Tyrrhenian treefrog *Hyla sarda* (Anura: Hylidae): adding pieces to the puzzle of Corsica-Sardinia biota.. Biol J Linn Soc.

[69] Barr TC, Holsinger JR (1985). Speciation in cave faunas.. Annu Rev Ecol Syst.

[70] Steinfartz S, Mutz T, Grossenbacher, Thiesmeier B (1998). Mertensiella luschani (Steindachner, 1891)- Lykischer Salamander, Kleinasiatischer Salamander.. Handbuch der Reptilien und Amphibien Europas, vol 4/I: Schwanzlurche, Wiesbaden.

[71] Verovnik R, Sket B, Trontelj P (2004). Phylogeography of subterranean and surface populations of water lice *Asellus aquaticus* (Crustacea: Isopoda).. Mol Ecol.

[72] Irwin DE (2002). Phylogeographic breaks without geographic barriers to gene flow.. Evolution.

[73] Johannesen J, Johannesen B, Griebeler EM, Baran I, Tunc MR (2006). Distortion of symmetrical introgression in a hybrid zone: evidence for a locus-specific selection and uni-directional range expansion.. J Evolut Biol.

[74] Liebgold EB, Brodie ED, Cabe PR (2010). Female philopatry and male-biased dispersal in a direct-developing salamander, *Plethodon cinereus*.. Mol Ecol.

[75] Wake DB, Salvador A, Alonso-Zarazafa MA (2005). Taxonomy of the plethodontid salamander genus *Hydromantes* (Caudata: Plethodontidae).. Amphibia-Reptilia.

[76] Speynbroeck J, Beukema W, Crochet P-A (2010). A tentative species list of the European herpetofauna (Amphibia and Reptilia)- an update.. Zootaxa.

[77] Welsh HH, Droege S (2001). A case for using plethodontid salamanders for monitoring biodiversity and ecosystem integrity of North American forests.. Cons Biol.

[78] Henle K, Dick D, Harpke A, Kühn I, Schweiger O (2008). Climate change impacts on European amphibians and reptiles.. Biodiversity and climate change: Reports and guidance developed under the Bern Convention.

[79] Bernini F, Doria G, Razzetti E, Sindaco R (2006). Atlante degli anfibi e rettili d'Italia..

[80] Lanza B, Bartolo G., Muzzetto G. (1991). Note faunistiche sulle grotte di Samugheo di Asuni, in particolare sul geotritone *Speleomantis imperialis*..

