# Supplementary material for: Phylogeography of Sardinian Cave Salamanders (Genus Hydromantes) Is Mainly Determined by Geomorphology
Source: PLoS One. 2012 Mar 12;7(3):e32332. doi: 10.1371/journal.pone.0032332 (PMC3299655; doi:10.1371/journal.pone.0032332)
Supplement: Information S1 — Problems relative to dating estimates for our data. (DOC) [file pone.0032332.s001.doc]

**Supporting Information S1:**

Three divergent time estimates of the split between American and European *Hydromantes* are currently available (Mueller, 2006, Carranza et al., 2007, Vieites et al., 2007). These estimates differ by at least 20 mya (60 mya, 13.5 mya, 41 mya, respectively) and are mostly the result of the use of one single vertebra fossil (Venczel and Sanchíz, 2005), which is by some accepted to belong to *Hydromantes* (Mueller, 2006, Vieites et al, 2007) and by other not (Carranza et al., 2007) and of different species sampling (Mueller, 2006, Vieites et al, 2007). Due to the lack of a better fossil record and to the use of a single rapidly evolving marker, performing the most accurate dating analysis based on our data is currently not possible. We therefore focused our work only on the geographical and geological factors that may represent a barrier to gene flow within and among Sardinian *Hydromantes* species, and not on the timing of the cladogenetic events.

Nonetheless, we attempted to address the appropriateness of the three alternative divergence-time estimates referred above by using these estimates to compute the median rate of molecular evolution at the cytochrome *b*, and comparing it to the average rate for all plethodontid salamanders presented in Table 2 of Mueller (2006) (0.62 ± 0.16 substitutions per nucleotide site per 100 mya), which was obtained considering a time of divergence between American and European *Hydromantes* 60 mya (95% CI, 48-73; Mueller, 2006). To compute the rates of evolution, we performed a Bayesian dating analysis using the BEAST v1.6.1 software package (Drummond and Rambaut, 2007) with five chains of 10 million generations each, sampling trees every 1000th generation. We used the ML tree obtained as described in the Methods section of our manuscript and its corresponding model of evolution as a starting tree. The Yule model of speciation was used for this analysis, as suggested by the tutorial for multiple species analysis. Log likelihood scores were graphed in Tracer and chain runs were checked for convergence. A Maximum Clade Credibility (MCC) tree was obtained combining the remaining trees after excluding the first 5000 trees for each chain. Median rates of molecular evolution were calculated, after removing the values for *Ensatina* and *Salamandra*, for the entire gene (all codon positions included) as in Mueller (2006) and were obtained from the output file of the MCC tree. We found that the use of 13.5 mya to calibrate the split between American and European *Hydromantes* (Carranza et al., 2007) results in a median rate of evolution (0.71 substitutions per nucleotide site per 100 mya) that best conforms to the average rate published by Mueller (2006). Rates obtained by applying the other two estimates were much lower than the published range of rates of molecular evolution at the cytochrome *b*. Therefore, we can conclude that a calibration of 13.5 mya seems more plausible because it brings the rates of molecular evolution closer to average published rate for this gene in plethodontids. However, any conclusion from results using this methodology should be taken with precaution. Given the use of all codon positions, no distinction is made between synonymos and nonsynonymous substitution rates, therefore results could be biased by variations in selective pressure. In order to overcome this limitation, a wide comparative study on plethodontid salamanders using rates of evolution based on synonymous substitution only (considered to be selectively neutral) should be performed.

**References:**

Carranza S, Romano A, Arnold EN, Sotgiu G (2007) Biogeography and evolution of European cave salamanders, *Hydromantes* (Urodela: Plethodontidae), inferred from mtDNA sequences. J. Biogeogr. 35: 724- 738.

Drummond AJ, Rambaut A (2007) BEAST: Bayesian evolutionary analysis by sampling trees. BMC Evol Biol **7**: 214.

Mueller RL (2006) Evolutionary rates, divergence dates, and the performance of mitochondrial genes in Bayesian Phylogenetic Analysis. Syst. Biol. 55: 289-300.

# Venczel M, Sanchíz B (2005) A fossil plethodontid salamander from the Middle Miocene of Slovakia (Caudata, Plethodontidae). Amphibia-Reptilia 26: 408-411.

Vieites DR, Min M-S, Wake DB (2007) Rapid diversification and dispersal during periods of global warming by plethodontid salamanders. Proc Natl Acad Sci 104: 19903- 19907.
