# Supplementary material for: Phylogeography of Sardinian Cave Salamanders (Genus Hydromantes) Is Mainly Determined by Geomorphology
Source: PLoS One. 2012 Mar 12;7(3):e32332. doi: 10.1371/journal.pone.0032332 (PMC3299655; doi:10.1371/journal.pone.0032332)
Supplement: Information S2 — Genetic p-distances among species (a) and intraspecific clades (b) of Italian Hydromantes . Average p-distances (minimum and maximum p-distances in parenthesis) based on 511 bp of the cytochrome b gene. Numbers with each species refer to clades as in Figure 2. (DOC) [file pone.0032332.s002.doc]

**Supporting information S2. Genetic p-distances among species (a) and intraspecific clades (b) of Italian *Hydromantes***. Average p-distances (minimum and maximum p-distances in parenthesis) based on 511 bp of the cytochrome *b* gene. In (b), numbers next to each species refer to clades as in Figure 2.

a)

|  | ***H. flavus*** | ***H. supramontis*** | ***H. sarrabusensis*** | ***H. imperialis*** | ***H. genei*** | ***H. ambrosii*** | ***H. italicus*** | ***H. strinatii*** |
| --- | --- | --- | --- | --- | --- | --- | --- | --- |
| ***H. flavus*** | - |  |  |  |  |  |  |  |
| ***H. supramontis*** | 0.093  (0.069-0.117) | - |  |  |  |  |  |  |
| ***H. sarrabusensis*** | 0.093  (0.081-0.110) | 0.086  (0.080-0.096) | - |  |  |  |  |  |
| ***H. imperialis*** | 0.093  (0.075-0.111) | 0.098  (0.076-0.129) | 0.099  (0.084-0.113) | - |  |  |  |  |
| ***H. genei*** | 0.149  (0.133-0.166) | 0.155  (0.123-0.174) | 0.147  (0.137-0.154) | 0.156  (0.141-0.178) | - |  |  |  |
| ***H. ambrosii*** | 0.136  (0.125-0.147) | 0.146  (0.133-0.157) | 0.134  (0.130-0.139) | 0.129  (0.116-0.143) | 0.152  (0.135-0.166) | - |  |  |
| ***H. italicus*** | 0.132  (0.123-0.143) | 0.138  (0.129-0.143) | 0.124  (0.121-0.125) | 0.131  (0.118-0.142) | 0.148  (0.139-0.162) | 0.051  (0.045-0.059) | - |  |
| ***H. strinatii*** | 0.136  (0.117-0.160) | 0.139  (0.121-0.157) | 0.131  (0.125-0.137) | 0.143  (0.125-0.158) | 0.139  (0.119-0.158) | 0.080  (0.065-0.089) | 0.086  (0.078-0.096) | - |

b)

|  | ***H. flavus 2*** | ***H. supramontis 2*** | ***H. imperialis 2*** | ***H. imperialis 3*** | ***H. imperialis 4*** | ***H. imperialis 5*** | ***H. imperialis 6*** | ***H. genei 2B*** | ***H. genei 3B*** | ***H. genei 4A*** | ***H. genei (2B+3B)= genei B*** |
| --- | --- | --- | --- | --- | --- | --- | --- | --- | --- | --- | --- |
| ***H. flavus 1*** | 0.041  (0.034-0.046) |  |  |  |  |  |  |  |  |  |  |
| ***H. supramontis 1*** |  | 0.060  (0.053-0.067) |  |  |  |  |  |  |  |  |  |
| ***H. imperialis 1*** |  |  | 0.050  (0.040-0.064) | 0.042  (0.038-0.050) | 0.052  (0.044-0.062) | 0.036  (0.028-0.046) | 0.033  (0.022-0.042) |  |  |  |  |
| ***H. imperialis 2*** |  |  |  |  |  |  | 0.049  (0.042-0.054) |  |  |  |  |
| ***H. imperialis 3*** |  |  | 0.027  (0.022-0.032) |  |  |  | 0.037  (0.034-0.038) |  |  |  |  |
| ***H. imperialis 4*** |  |  | 0.040  (0.034-0.048) | 0.041  (0.040-0.042) |  |  | 0.043  (0.040-0.046) |  |  |  |  |
| ***H. imperialis 5*** |  |  | 0.043  (0.038-0.048) | 0.033  (0.030-0.038) | 0.041  (0.036-0.046) |  | 0.037  (0.034-0.038) |  |  |  |  |
| ***H. genei 1A*** |  |  |  |  |  |  |  | 0.051  (0.037-0.059) | 0.044  (0.037-0.047) | 0.088  (0.082-0.092) | 0.050 (0.037-0.059) |
| ***H. genei 2B*** |  |  |  |  |  |  |  |  | 0.030 (0.027-0.035) | 0.083 (0.080-0.086) |  |
| ***H. genei 3B*** |  |  |  |  |  |  |  |  |  | 0.083 (0.082-0.084) |  |
| ***H. genei 4A*** |  |  |  |  |  |  |  |  |  |  | 0.083 (0.080-0.086) |
