# Supplementary material for: Phylogeography of Sardinian Cave Salamanders (Genus Hydromantes) Is Mainly Determined by Geomorphology
Source: PLoS One. 2012 Mar 12;7(3):e32332. doi: 10.1371/journal.pone.0032332 (PMC3299655; doi:10.1371/journal.pone.0032332)
Supplement: Information S3 — Intraspecific haplotype networks distances. Minimum number of steps (absolute genetic distances) to connect intraspecific single haplotype networks. (DOC) [file pone.0032332.s003.doc]

**Supporting information S3. Intraspecific haplotype networks distances.** Minimum number of steps (absolute genetic distances) to connect intraspecific single haplotype networks. Numbers next to each species refer to separate networks as in Figures 5 and 6.

|  | ***H. flavus* I** | ***H. supramontis* I** | ***H. imperialis* I** | ***H. imperialis* II** | ***H. imperialis* III** | ***H. imperialis* IV** | ***H. genei* I** | ***H. genei* II** | ***H. genei* III** |
| --- | --- | --- | --- | --- | --- | --- | --- | --- | --- |
| ***H. flavus* II** | 16 | **-** | **-** | **-** | **-** | **-** | **-** | **-** | **-** |
| ***H. supramontis* II** | **-** | 32 | **-** | **-** | **-** | **-** | **-** | **-** | **-** |
| ***H. imperialis* II** | **-** | **-** | 19 | **-** | **-** | **-** | **-** | **-** | **-** |
| ***H. imperialis* III** | **-** | **-** | 15 | 17 | **-** | **-** | **-** | **-** | **-** |
| ***H. imperialis* IV** | **-** | **-** | 17 | 12 | 18 | **-** | **-** | **-** | **-** |
| ***H. imperialis* V** | **-** | **-** | 11 | 13 | 18 | 11 | **-** | **-** | **-** |
| ***H. genei* I** | **-** | **-** | **-** | **-** | **-** | **-** | **-** | **-** | **-** |
| ***H. genei* II** | **-** | **-** | **-** | **-** | **-** | **-** | 39 | **-** | **-** |
| ***H. genei* III** | **-** | **-** | **-** | **-** | **-** | **-** | 18 | 41 | **-** |
| ***H. genei* IV** | **-** | **-** | **-** | **-** | **-** | **-** | 14 | 38 | 18 |
