# Supplementary material for: Phylogeography of Sardinian Cave Salamanders (Genus Hydromantes) Is Mainly Determined by Geomorphology
Source: PLoS One. 2012 Mar 12;7(3):e32332. doi: 10.1371/journal.pone.0032332 (PMC3299655; doi:10.1371/journal.pone.0032332)
Supplement: Information S4 — Results of the SAMOVA/AMOVA analyses. Intraspecific genetic structure for each Hydromantes species on the base of the within and among population genetic variance, the geographic location and sampling site. This was not tested for H. sarrabusensis, as only one haplotype network was recovered for this species. Populations belonging to distinct clusters (gene pools) are indicated in parenthesis with numbers referring to populations as in Table 1. FST, FCT, and FSC indicate the proportion of total variance among populations, among groups, and among populations within groups, respectively. * indicates significant p-values<0.05. (DOC) [file pone.0032332.s004.doc]

**Supporting information S4. Results of the SAMOVA/AMOVA analyses.** Intraspecific genetic structure for each *Hydromantes* species on the base of the within and among population genetic variance, the geographic location and sampling site. This was not tested for *H. sarrabusensis*, as only one haplotype network was recovered for this species. Populations belonging to distinct clusters (gene pools) are indicated in parenthesis with numbers referring to populations as in Table 1. *F_ST_*, *F_CT_*, and *F_SC_* indicate the proportion of total variance among populations, among groups, and among populations within groups, respectively. * indicates significant p-values <0.05.

| **Species** |  | **Variance** | **% of variation** | ***F* statistics** | **P-value** |
| --- | --- | --- | --- | --- | --- |
| ***H. flavus*** |  |  |  |  |  |
| One gene pool | Among populations | 4.46467 | 95.58 | *Fst*: 0.95579 | 0.000* |
|  | Within populations | 0.20652 | 4.42 |  |  |
| Two gene pools (pops 1, 2, 3, 4, 5, 6, 7; pops 8, 9) | Among groups | 8.41562 | 88.55 | *Fct*: 0.88547 | 0.026* |
|  | Among populations within groups | 0.88197 | 9.28 | *Fsc*: 0.81027 | 0.000* |
|  | Within populations | 0.20652 | 2.17 | *Fst*: 0.97827 | 0.000* |
| Two gene pools- according to separated calcareous areas (pops 1, 2, 3, 4, 5, 6, 7, 8; pop 9) | Among groups | 4.89702 | 58.63 | Fct: 0.58632 | 0.104 |
|  | Among populations within groups | 3.24856 | 38.90 | *Fsc*: 0.94023 | 0.000* |
|  | Within populations | 0.20652 | 2.47 | *Fst*: 0.97527 | 0.000* |
| ***H. supramontis*** |  |  |  |  |  |
| One gene pool | Among populations | 10.12678 | 98.67 | *Fst*: 0.98669 | 0.000* |
|  | Within populations | 0.13662 | 1.33 |  |  |
| Two gene pools (pops 29, 30, 31, 32, 33, 34, 35; pops 36, 37) | Among groups | 14.3280 | 76.82 | *Fct*: 0.76816 | 0.039* |
|  | Among populations within groups | 4.18773 | 22.45 | *Fsc*: 0.96841 | 0.000* |
|  | Within populations | 0.13662 | 0.73 | *Fst*: 0.99268 | 0.000* |
| Three gene pools (pops 29, 31, 32, 33; pops 30, 34, 35; pops 36, 37) | Among groups | 10.34925 | 80.20 | *Fct*: 0.80200 | 0.000* |
|  | Among populations within groups | 2.41846 | 18.74 | *Fsc*: 0.94653 | 0.000* |
|  | Within populations | 0.13662 | 1.06 | *Fst*: 0.98941 | 0.000* |
| Three gene pools- according to separated calcareous areas (pops 29, 30, 31, 32, 34, 35; pops 36, 37; pop 33) | Among groups | 11.12550 | 75.01 | *Fct*: 0.75011 | 0.006* |
|  | Among populations within groups | 3.56965 | 24.07 | *Fsc*: 0.96314 | 0.000* |
|  | Within populations | 0.13662 | 0.92 | *Fst*: 0.99079 | 0.000* |
| ***H. imperialis*** |  |  |  |  |  |
| One gene pool | Among populations | 7.19085 | 94.95 | *Fst*: 0.94949 | 0.000* |
|  | Within populations | 0.38250 | 5.05 |  |  |
| Two gene pools (pops 10, 11, 12, 13, 14, 15, 16, 17, 18, 19, 20, 21, 24, 27, 28; pops 22, 23, 25, 26) | Among groups | 5.69648 | 47.94 | *Fct*: 0.47938 | 0.000* |
|  | Among populations within groups | 5.80402 | 48.84 | *Fsc*: 0.93817 | 0.000* |
|  | Within populations | 0.38250 | 3.22 | *Fst*: 0.96781 | 0.000* |
| Three gene pools (pops 10, 11, 12; pops 13, 14, 15, 16, 17, 18, 19, 20, 21, 24, 27, 28; pops 22, 23, 25, 26) | Among groups | 6.57609 | 60.46 | *Fct*: 0.60458 | 0.000* |
|  | Among populations within groups | 3.91857 | 36.03 | *Fsc*: 0.91107 | 0.000* |
|  | Within populations | 0.38250 | 3.52 | *Fst*: 0.96483 | 0.000* |
| Four gene pools (pops 10, 11, 12; pops 13, 14, 17, 18, 20, 24, 27, 28; pops 15, 16, 19, 21; pops 22, 23, 25, 26) | Among groups | 6.05378 | 65.14 | *Fct*: 0.65145 | 0.000* |
|  | Among populations within groups | 2.85651 | 30.74 | *Fsc*: 0.88191 | 0.000* |
|  | Within populations | 0.38250 | 4.12 | *Fst*: 0.95884 | 0.000* |
| Five gene pools (pops 10, 11, 12, 21; pop 19; pops 13, 14, 17, 18, 20, 24, 27, 28; pops 22, 23, 25, 26) | Among groups | 6.94988 | 73.91 | *Fct*: 0.73906 | 0.000* |
|  | Among populations within groups | 2.07136 | 22.03 | *Fsc*: 0.84412 | 0.000* |
|  | Within populations | 0.38250 | 4.07 | *Fst*: 0.95932 | 0.000* |
| Six gene pools (pops 10, 11, 12, 21; pops 13, 14, 17, 18, 20, 24; pops 15, 16; pop 19; pops 22, 23, 25, 26; pops 27, 28) | Among groups | 6.77485 | 78.03 | *Fct*: 0.78025 | 0.000* |
|  | Among populations within groups | 1.52556 | 17.57 | *Fsc*: 0.79954 | 0.000* |
|  | Within populations | 0.38250 | 4.41 | *Fst*: 0.95595 | 0.000* |
| Twelve gene pools - according to separated calcareous areas (pops 10, 11, 12; pops 13, 14; pops 15, 16; pops 17, 18, 19; pop 20; pop 21; pops 22, 23; pop 24; pop 25, pop 26; pop 27; pop 28) | Among groups | 4.68445 | 59.28 | *Fct*: 0.59284 | 0.000* |
|  | Among populations within groups | 2.83470 | 35.87 | *Fsc*: 0.88111 | 0.000* |
|  | Within populations | 0.38250 | 4.84 | *Fst*: 0.95159 | 0.000* |
| ***H. genei*** |  |  |  |  |  |
| One gene pool | Among populations | 9.80543 | 95.64 | *Fst*: 0.95643 | 0.000* |
|  | Within populations | 0.44667 | 4.36 |  |  |
| Two gene pools (pops 40, 41, 42, 43, 45, 46, 47, 48; pop 44) | Among groups | 13.1373 | 62.53 | *Fct*: 0.62527 | 0.106 |
|  | Among populations within groups | 7.42667 | 35.35 | *Fsc*: 0.94327 | 0.000* |
|  | Within populations | 0.44667 | 2.13 | *Fst*: 0.97874 | 0.000* |
| Two gene pools – according to groups called *genei* A and B (pops 40, 41, 42, 43, 44; pops 45, 46, 47, 48) | Among groups | 6.45879 | 50.03 | *Fct*: 0.50029 | 0.000* |
|  | Among populations within groups | 6.00458 | 46.51 | *Fsc*: 0.93076 | 0.000* |
|  | Within populations | 0.44667 | 3.46 | *Fst*: 0.96540 | 0.022* |
| Three gene pools (pops 40, 41, 42, 43; pop 44; pop 45, 46, 47, 48) | Among groups | 11.6811 | 83.43 | *Fct*: 0.83427 | 0.004* |
|  | Among populations within groups | 1.87385 | 13.38 | *Fsc*: 0.80751 | 0.000* |
|  | Within populations | 0.44667 | 3.19 | *Fst*: 0.96810 | 0.000* |
| Four gene pools (pops 40, 41, 42, 43; pop 44; pops 45, 46, 47; pop 48) | Among groups | 12.0954 | 88.70 | *Fct*: 0.88697 | 0.000* |
|  | Among populations within groups | 1.09473 | 8.03 | *Fsc*: 0.71022 | 0.000* |
|  | Within populations | 0.44667 | 3.28 | *Fst*: 0.96725 | 0.000* |
| Four gene pools - according to separated calcareous areas (pops 40, 41, 42, 43, 44; pops 45; pops 46, 47; pop 48) | Among groups | 3.33022 | 29.89 | *Fct*: 0.29886 | 0.144 |
|  | Among populations within groups | 7.36601 | 66.11 | *Fsc*: 0.94283 | 0.000* |
|  | Within populations | 0.44667 | 4.01 | *Fst*: 0.95991 | 0.000* |
